# Effect of pH on the Efficiency of Pyrogallol, Gallic Acid, and Alkyl Gallates in Trapping Methylglyoxal

**DOI:** 10.3390/molecules30153086

**Published:** 2025-07-23

**Authors:** Haria Hadjipakkou, Eftychia Pinakoulaki

**Affiliations:** Department of Chemistry, University of Cyprus, 1 Panepistimiou Avenue, Aglantzia, 2109 Nicosia, Cyprus; hadjipakkou.charia@ucy.ac.cy

**Keywords:** phenolics, *a*-dicarbonyls, trapping, liquid chromatography-mass spectrometry

## Abstract

Methylglyoxal (MGO) is a highly reactive *a*-dicarbonyl compound produced in foods and endogenously in humans and constitutes a predominant precursor of advanced glycation end products that contribute to the pathology of several diseases, including diabetes and neurodegenerative diseases. In this study, the efficiency of pyrogallol, gallic acid, ethyl, and propyl gallate in trapping MGO was investigated at pH 6.5 to 8.0. Pyrogallol was the most efficient MGO-trapping agent, followed by gallic acid, whereas the alkyl gallates were notably less efficient, particularly at slightly acidic and neutral pH. The increase of pH from slightly acidic to alkaline enhanced the MGO-trapping efficiency of all compounds, albeit to a different extent that correlated inversely to the p*K*_a_ of the most acidic -OH phenolic group, demonstrating the contribution of the deprotonated forms of the phenolic compounds in the enhanced reactivity towards MGO. The reaction products of pyrogallol, identified as the most efficient compound in MGO-trapping, were analyzed and characterized by liquid chromatography-mass spectrometry (LC-MS). Both mono-MGO and di-MGO conjugated adducts of pyrogallol were detected, with the mono-MGO adduct being dominant solely at acidic pH and the di-MGO pyrogallol adducts becoming prevalent at neutral and alkaline pH. Therefore, the pH was determined as a main factor that controls the reaction pathways of the phenolic compounds with MGO.

## 1. Introduction

Methylglyoxal (MGO) is a highly reactive *a*-dicarbonyl compound that is formed endogenously in humans and in carbohydrate and lipid-rich foods by the Maillard reaction and lipid oxidation [1,2,3]. MGO is a predominant precursor of advanced glycation end products (AGEs), a family of compounds that are products of nonenzymatic reactions between reducing sugars and proteins, lipids, or nucleic acids [4,5]. In fact, MGO is up to 20,000 times more potent as a precursor for AGEs formation compared to glucose. MGO reacts with the N-termini and lysine and arginine side chains of peptides and proteins and is also able to covalently modify DNA, thus leading to the formation of various AGEs [4,5]. Endogenous AGEs have been associated with the pathogenesis and progression of several diseases such as diabetes, atherosclerosis, osteoporosis, cancer, and neurodegenerative diseases. In addition, several studies implicated the dietary intake of AGEs in synergistically increasing the systemic AGE load and resulting in adverse health effects, although this has been challenged [4,6,7]. Some AGEs typically found in food include Nϵ-carboxymethyl-lysine, Nϵ-carboxyethyl-lysine, pyrraline, pentosidine, and imidazolium cross-link derived from methylglyoxal and lysine-lysine or from glyoxal and lysine-lysine [3]. Although these compounds and the Maillard reaction products in general contribute to the aroma, flavor, and color of foods, the nutritional value decreases [2,3]. Other adverse effects of MGO in foods include the formation of toxic compounds such as acrylamide and 4 (5)-methylimidazole [8,9].

During the last years, research efforts have been focusing on identifying compounds that can effectively scavenge MGO both in foods and in vivo, to inhibit the formation and accumulation of AGEs and other toxins. One family of compounds that has received particular attention is polyphenols [10,11,12]. Polyphenols are secondary plant metabolites that are known to exert many beneficial effects in human health through their antioxidant, anti-inflammatory, and anticancer activities [13,14], and their potential to scavenge *a*-dicarbonyls represents an additional aspect of their beneficial roles. Phenolic compounds that have been studied include phenolic acids, flavonoids, and stilbenes, which can trap the electrophilic MGO through an electrophilic substitution reaction to produce mainly mono- and di-MGO conjugated polyphenolic adducts. Some examples of flavonoids that exhibited significant MGO-trapping capacity at physiological conditions (pH 7.4 and 37 °C) include epigallocatechin-3-gallate [15], phloretin [16], genistein [17,18], quercetin [19,20], and epicatechin [21]. Recent studies have also identified flavonoids and alkaloids that effectively trap MGO and other carbonyl compounds at 100 °C and neutral pH, including mangiferin and synephrine alone or in combination with neohesperidin [22,23]. Comparative studies have been instrumental in identifying the structural features that are essential for the efficient trapping of MGO. There is consensus that the A ring is the active site of flavonoids, with the hydroxy group at C5 enhancing the trapping efficiency, while the hydroxyl groups on the B ring do not have a significant effect [24,25]. On the other hand, there are controversial views on the role of structural features of the C ring [24,25]. Except for the molecular structure, the pH of the reaction has been described as a factor contributing to the MGO-trapping efficiency of flavonoids. Although the majority of studies have been performed at physiological pH, it was reported that epigallocatechin-3-gallate loses its trapping efficacy under acidic conditions (pH ≤ 4) [15]. A subsequent study addressed the effect of pH on the reaction of naringenin with MGO and reported that the reaction rate increased with pH, proposing a base-catalyzed mechanism [26]. Taking into consideration that the effect of pH in the MGO-tapping efficiency has been studied only for members of the flavonoids group and that the pH of foods varies from acidic to basic (e.g., yogurt pH ~ 4, milk pH ~ 6.8, egg pH ~ 8.0), it’s imperative to extend pH studies to other subclasses of phenolic compounds. 

Gallic acid, which belongs to the group of phenolic acids and is ubiquitously found in plant species, as well as its decarboxylated derivative pyrogallol and its alkyl esters, ethyl and propyl gallate, the latter widely used as a food additive, were chosen for this study. The existing literature includes studies on the MGO-trapping efficiency of these compounds that have been performed only at pH 7.4. Pyrogallol is regarded as an efficient scavenger of MGO at pH 7.4, reported to trap 90% of MGO within 24 h incubation at 37 °C, while for gallic acid, the reported values for trapping of MGO are discrepant and vary from 14.9% to 82.7% for 24 h incubation [24,27]. For shorter incubation times, gallic acid was reported to trap 11.1% (at 1 h) and 28.5% of MGO (at 2 h) [27,28]. As far as alkyl gallates are concerned, methyl gallate trapped only 5% of MGO at pH 7.4 after 1 h incubation at 37 °C [28]. On the other hand, when the reaction of propyl gallate with MGO was performed at 100 °C and pH 7, propyl gallate trapped 77.5% of MGO after 30 min incubation, which was significantly higher compared to 15.2% reported for gallic acid under the same conditions [29]. 

In this work, we examined and compared the MGO-trapping efficiency of pyrogallol, gallic acid, ethyl, and propyl gallates at the pH range from 6.5 to 8.0, thus addressing conditions in slightly acidic to alkaline foods. Moreover, considering that foods may be processed and stored in a wide range of temperatures and that the vast majority of experiments in the related literature have been performed at 37 °C, we have also used 37 °C as the temperature for studying the MGO-trapping reactions in the present study to enable comparisons with the existing literature. Pyrogallol, which was identified as the most efficient compound in MGO-trapping in the overall pH studied, was further investigated, and the reaction products were separated and characterized by LC-MS to identify if different reaction pathways are enhanced under slightly acidic and basic conditions.

## 2. Results and Discussion

### 2.1. Effect of pH in Trapping of MGO by Pyrogallol, Gallic Acid, and Gallate Esters

**Figure 1:**
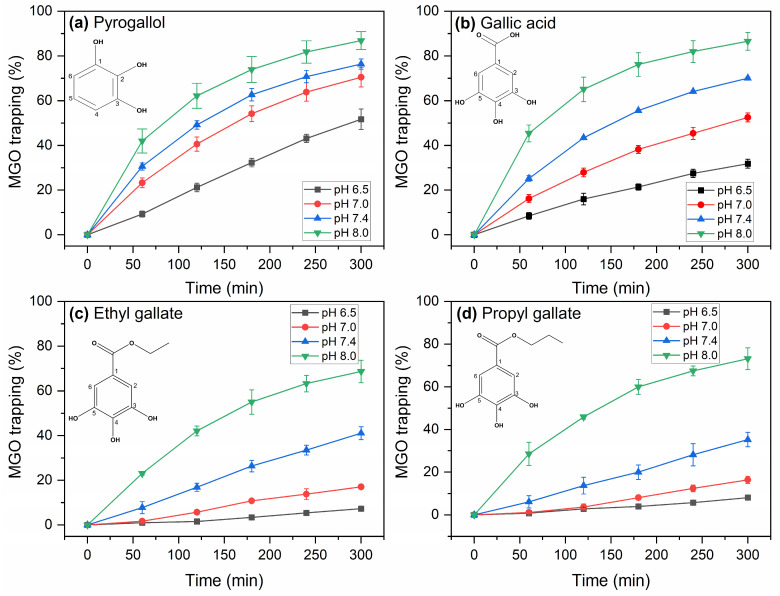
Trapping of MGO by pyrogallol (**a**), gallic acid (**b**), ethyl gallate (**c**), propyl gallate (**d**) at pH 6.5 (black squares), pH 7.0 (red circles), pH 7.4 (blue up-pointing triangles), and pH 8.0 (green down-pointing triangles) after incubation at 37 °C.

Figure 1 describes the trapping of MGO (expressed as % percent of trapped MGO) by pyrogallol (a), gallic acid (b), ethyl gallate (c) and propyl gallate (d) after incubation at 37 °C for 1, 2, 3, 4 and 5 h at pH 6.5, 7.0, 7.4 and 8.0. At slightly acidic pH (pH 6.5), pyrogallol trapped 9.3% of MGO at 1 h incubation at 37 °C, increasing to 51.7% within 5 h. At pH 7.0, MGO-trapping by pyrogallol increased to 23.3% and 70.5% at 1 h and 5 h incubation, respectively. Further increase was observed for pH 7.4, and at pH 8.0, the MGO-trapping reached 42% at 1h and 86.9% at 5 h. The corresponding trapping efficiency of gallic acid was lower compared to pyrogallol at pH 6.5 and 7.0, but became comparable at pH 7.4 and 8.0. In contrast, both ethyl and propyl gallate were significantly less efficient in trapping MGO at pH 6.5 and 7.0, reaching only ~8% and ~17%, respectively, at 5 h incubation. For both alkyl gallate esters, a significant increase was observed at slightly basic pH, and MGO trapping reached ~70% at 5 h incubation at pH 8.0. We note that the reported MGO-trapping data include the pH 6.5 to 8.0 range, since below pH 6, the MGO-trapping efficiency of the examined compounds was significantly diminished. The data for all phenolic compounds at the different pHs and incubation times are included in Supplementary Materials (Table S1).

To describe the effect of the factors that were investigated in this work (phenolic compound, pH, and incubation time) and their interactions, three-way ANOVA was employed, and the results are presented in Supplementary Materials (Figures S1–S3). The results over the pH 6.5 to 8.0 range highlighted pyrogallol as the most efficient MGO trapping agent among the four compounds examined, followed by gallic acid, while the alkyl gallates were notably less efficient. Statistical analysis also underscored the pH as a dominant factor for the reactivity of the phenolic compounds towards MGO, with a gradual increase in the trapping of MGO when varying the pH from 6.5 to 7.0 and 7.4 and a more substantial increase at pH 8.0, especially for the alkyl gallates.

The compounds studied herein are benzenetriol derivatives that trap the electrophilic MGO through an electrophilic substitution reaction. Overall, our results suggest that pyrogallol is the most efficient compound in MGO-trapping, although gallic acid is marginally less efficient, especially in alkaline conditions. Sang and coworkers [24] reported that pyrogallol efficiently trapped MGO at pH 7.4, in contrast to gallic acid, and suggested that the C5 of pyrogallol was the active position for trapping MGO, thus rationalizing the inefficiency of gallic acid. Lo et al. [28] used computational chemistry calculations to show that the symmetric C4 and C6 of pyrogallol had the highest negative electron charge and were the target sites for MGO-trapping, while the addition of the electron-withdrawing carboxyl group in gallic acid made the benzene ring less reactive to nucleophilic attack. However, taking into account that in the pH 6.5 to 8.0 range studied herein, the carboxylic group is in fact deprotonated (p*K*_a (COOH)_ is 4.0), its electron-withdrawing effect will be diminished [30]. Therefore, as we observed experimentally, gallic acid is active in trapping MGO, albeit with rather lower efficiency compared to pyrogallol. Our results are also consistent with recent work demonstrating that gallic acid effectively inhibited protein glycation and aggregation induced by MGO via the synergy of trapping MGO, scavenging free radicals, and interacting with proteins [31]. In contrast to gallic acid, ethyl and propyl gallates exhibited a strikingly limited MGO-trapping ability, particularly at slightly acidic and neutral pH, that cannot be rationalized by the electronic effects of the alkyl ester groups on the benzene ring and is therefore attributed to increased steric hindrance, in agreement with what was previously proposed for methyl gallate [28]. The opposite trend was reported in the comparison of the MGO-trapping efficiency of propyl gallate and gallic acid when the reactions were performed at 100 °C and pH 7, with propyl gallate trapping 77.5% of MGO after 30 m incubation, while only 15.2% of MGO was trapped by gallic acid. Therefore, at 100 °C, propyl gallate was determined as significantly more efficient in MGO-trapping compared to gallic acid [29]. Thus, for alkyl esters, steric hindrance is suggested to be a dominant factor, imposing a high energy barrier on the reaction and rendering the compounds inefficient in MGO-trapping at lower temperatures and acidic to neutral pH, while at higher reaction temperatures this barrier is overcome.

The effect of pH on the MGO-trapping efficiency of all compounds examined is evident, as slightly acidic pH significantly decreases MGO-trapping ability and alkaline conditions enhance it. To interpret the effect of pH, its influence on the reactivities of both the phenolic compounds and MGO should be considered. The benzenetriols studied in this work have the same *vic*-trihydroxyl group; however, the differences in their molecular structure dictate distinct properties for the hydroxyl groups. Specifically, the p*K*_a_ for the deprotonation of the most acidic -OH group is 9.1 for pyrogallol, 8.7 for gallic acid, and 7.9 for the alkyl gallates [32,33]. The p*K*_a_ values appear to correlate inversely to the extent to which the increase of pH from 6.5 to 8.0 affects the efficiency of each compound in MGO-trapping, since we observed a smaller effect for pyrogallol compared to gallic acid and a pronounced effect for alkyl gallates at pH 8.0. This confirms that the deprotonated forms of the phenolic compounds have enhanced reactivity towards MGO, as the deprotonation of the most acidic -OH group leads to an increase of the partial electron density at the benzene ring due to the interconversion of enolate and keto forms. This way, while pyrogallol is more efficient than gallic acid due to its molecular structure at pH 6.5, when the deprotonated forms are virtually absent, their efficiencies in MGO-trapping become similar at pH 8.0 because the population of the more reactive deprotonated form is higher for gallic acid (17% for gallic acid and 7% for pyrogallol). The MGO-trapping efficiency of alkyl gallates is significantly increased at pH 8.0, since at pH 8.0 more than half of the population is deprotonated. Therefore, while the alkyl gallates are inefficient in MGO-trapping at pH 6.5, the deprotonation of the -OH group at pH 8.0 results in considerable enhancement of their activity towards MGO scavenging. However, the effect of pH on the phenolic compounds alone cannot account for our observations. The reactivity of MGO is also raised with increasing pH. MGO exists in equilibrium populations of monohydrated and dehydrated (unreactive) forms in aqueous solutions that can be dehydrated by base-catalytic action to yield the most reactive electrophilic aldehyde form (Supplementary Materials, Figure S4) [26,34]. Overall, our data and interpretations are in agreement with the base catalytic reaction proposed for the trapping of MGO by naringenin [26], rather than the two-step mechanism reported for the trapping of MGO by flavonoid aglycones in acidic conditions based on computational chemistry calculations [35].

It is also important to note that while many flavonoids have shown very high efficiencies in trapping MGO and other carbonyl compounds [15,16,17,18,19,20,21,22,23,24], their limited water solubilities often restrict potential food applications. However, gallic acid and its decarboxylated derivative, pyrogallol, present a significant advantage due to their high solubility and effectiveness in MGO-trapping that increases under slightly alkaline conditions. In fact, while pyrogallol is more efficient in trapping MGO compared to gallic acid at slightly acidic and neutral conditions, their efficiencies become comparable at alkaline conditions. Beyond this, gallic acid is well-known for its potent antioxidant and anti-inflammatory properties and has also been demonstrated to attenuate in vivo the expression of the receptor for AGEs (RAGE), suggesting considerable therapeutic potential and combined benefits for food applications [36,37]. On the other hand, the synthetic alkyl gallate esters that are designed for lipid-rich foods and were reported to be efficient MGO scavengers during thermal food processing [29] would not effectively trap MGO during storage in slightly acidic to neutral pH foods, based on our experiments.

### 2.2. MGO Adducts of Pyrogallol at pH 6.5 to 8.0 by LC-MS Analysis

**Figure 2:**
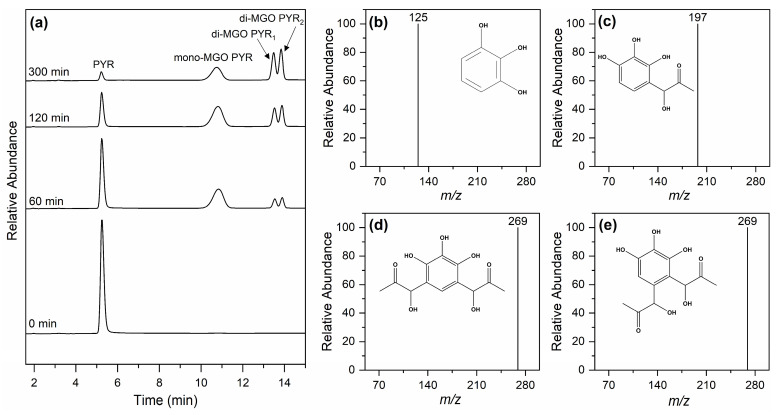
LC-MS chromatograms of the reaction of pyrogallol with MGO at pH 7.0 and 37 °C at the indicated incubation times (0, 60, 120, and 300 min) (**a**). The chromatograms were vertically translated for clarity. The peak with t_R_ of 5.2 min corresponds to the molecular ion at *m*/*z* 125 [M − H]^−^ (**b**), the peak with t_R_ of 10.8 min to the molecular ion at *m*/*z* 197 [M − H]^−^ (**c**), and the peaks with t_R_ of 13.4 and 13.8 min, both correspond to the molecular ion at *m*/*z* 269 [M − H]^−^ (**d**,**e**).

Considering the overall data in the pH 6.5–8.0 range, pyrogallol was identified as the most efficient compound in MGO-trapping, and thus, we further investigated its reaction mechanism by the analysis of the reaction products utilizing LC-MS. The chromatograms of the reaction of pyrogallol with MGO at neutral pH at 1, 2, 3, and 5 h of incubation are shown in Figure 2a. The chromatogram corresponding to the time immediately after mixing of MGO with pyrogallol included a peak with t_R_ of 5.2 min that is due to pyrogallol, as confirmed by its molecular ion at *m*/*z* 125 [M − H]^−^ (Figure 2b). After 1 h of incubation, three new peaks appeared. The peak with t_R_ 10.8 min had a molecular ion at *m*/*z* 197 [M − H]^−^ (Figure 2c), thereby it was 72 mass units higher than that of pyrogallol, indicating that it corresponds to the mono-MGO conjugated adduct of pyrogallol (mono-MGO PYR). Two additional peaks with t_R_ = 13.4 and 13.8 min had a molecular ion at *m*/*z* 269 [M − H]^−^ (Figure 2d,e), which is an additional 72 mass units higher and can therefore be attributed to the di-MGO conjugated adduct of pyrogallol (di-MGO PYR). This indicates that two distinct di-MGO PYR adducts were formed upon the MGO-pyrogallol reaction. The time dependence of the chromatographs allowed us to monitor the formation of the products, revealing that in the initial phase of the reaction, the mono-MGO PYR adduct was mainly formed, and the di-MGO PYR adducts became prevalent at longer incubation times at pH 7.0. The proposed structures of the mono-MGO PYR and di-MGO PYR adducts are depicted in Figure 2.

**Figure 3:**
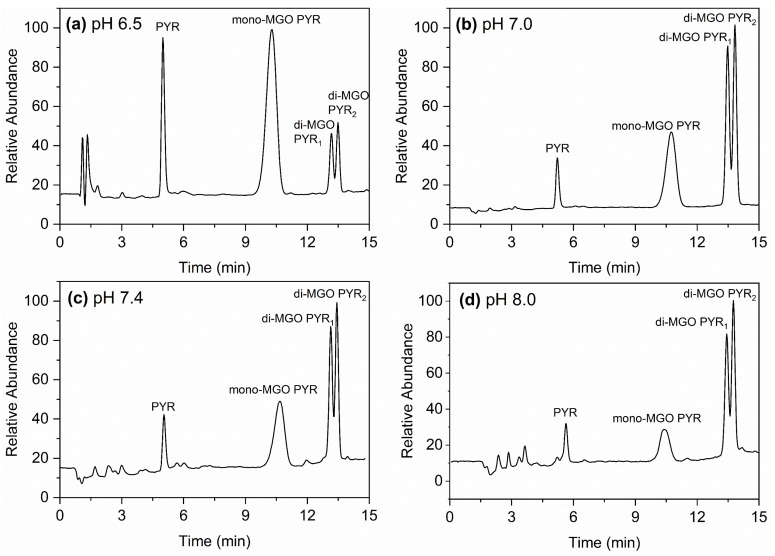
LC-MS chromatograms of the reaction of pyrogallol with MGO at pH 6.5 (**a**), pH 7.0 (**b**), pH 7.4 (**c**), and pH 8.0 (**d**) after incubation at 37 °C.

To examine whether the pH could be a factor that determines different pathways in the reaction of pyrogallol with MGO, LC chromatograms were obtained at prolonged incubation times in the pH 6.5–8.0 range. It is noted that since the reaction rate was significantly lower at acidic pH and higher at basic conditions, chromatograms correspond to the reaction time when the mono-/di- adduct ratio became constant in each pH (i.e., for pH 6.5, this occurs at 12 h incubation). Figure 3 shows the LC-MS chromatograms of the reaction of pyrogallol with MGO at pH 6.5 (a), 7.0 (b), 7.4 (c), and 8.0 (d). The four peaks present at all chromatograms correspond to unreacted pyrogallol, the mono-MGO PYR adduct, and the two di-MGO PYR products, as described previously in Figure 2. Notably, the formation of the mono-MGO PYR was favored over the di-MGO PYR adducts at pH 6.5, with a ratio of 4:1, while at pH 7.0, 7.4 and 8.0 the di-MGO PYR adducts became the main products with gradual increasing population as pH was raised, so that at pH 8.0 the mono-MGO PYR: di-MGO PYR ratio reached 1:4. The ratio of the two di-MGO adducts was also affected by the pH to some extent, with the population of the di-MGO PYR_2_ adduct (t_R_ = 13.8 min) increasing over the di-MGO PYR_1_ adduct (t_R_ = 13.4 min) with increasing pH; the di-MGO PYR_1_: di-MGO PYR_2_ ratio changed from 1:1.1 at pH 6.5 to 1:1.4 at pH 8.0.

**Figure 4:**
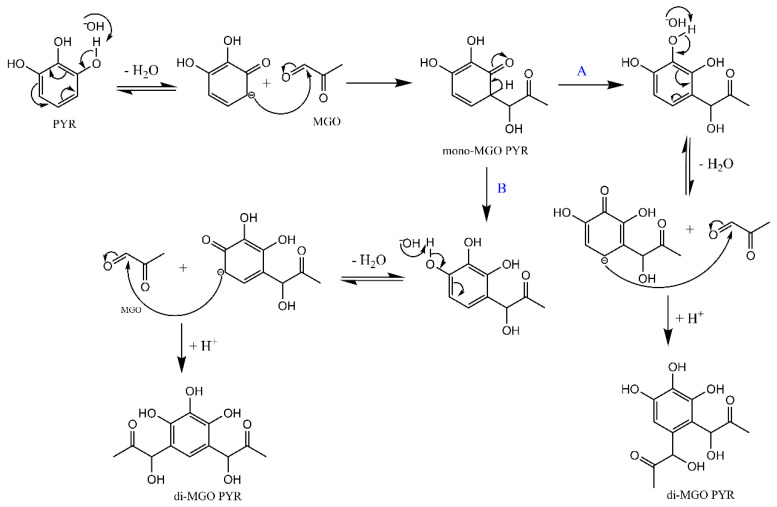
Proposed mechanism for the reaction of pyrogallol with MGO.

The characterization of the products of the reaction between pyrogallol and MGO allowed us to further evaluate the effect of pH on the mechanism of MGO scavenging. Our results demonstrated that the formation of mono-MGO PYR and di-MGO PYR is not only time-dependent, but more importantly, pH-dependent. Specifically, at slightly acidic pH, the mono-MGO PYR was the main product, while at neutral to basic pH, the di-MGO PYR became prevalent. Therefore, the pH affected the reactivity of both pyrogallol and its mono-MGO PYR adduct, but in a distinct way. It should be noted that a previous study on the MGO-trapping efficiency of pyrogallol reported the formation of only one di-MGO PYR product and associated the amounts of the mono-MGO PYR and di-MGO PYR that were formed in the reaction to the molar ratio of the reactants [24]. This was later challenged by Lund and coworkers [26], who suggested that the formation of products in the reactions of polyphenols with MGO is only affected by the reaction time, with the mono-MGO polyphenol adducts being formed initially and di-MGO adducts becoming the main products at prolonged incubation times. The experiments reported herein highlight the pH as a key factor that determines the reaction products, revealing the presence of alternative pathways in the reaction of pyrogallol with MGO that lead to the formation of the mono-MGO PYR and the two distinct di-MGO PYR adducts. Figure 4 depicts the proposed mechanism for the reaction. Both A and B pathways are less active at slightly acidic pH and become more active as the pH increases.

In conclusion, this work highlights the pH as a dominant factor in the reaction of phenolic compounds with MGO. The comparative study of the four structurally related benzenetriols revealed that the effect of pH on the reactivity of the benzene ring inversely correlated to the p*K*_a_ of the most acidic phenolic -OH group, therefore emphasizing the contribution of the deprotonated forms in MGO trapping. The variation of pH from slightly acidic to alkaline increased both the reactivity of the benzene ring of the phenolic compounds and the dehydration of MGO, making it more electrophilic, thus enhancing the efficiency of the electrophilic substitution reaction. In addition, the formation of the mono- and di-MGO conjugated pyrogallol products was pH dependent, indicating that the pH distinctly affects the reactivity of the phenolic reactant and its mono-MGO conjugated adduct towards MGO. Considering that the reactions of nucleophilic amino acids with *a*-dicarbonyl compounds are also pH-dependent [38], comparative and competitive pH studies are required to elucidate the potential of polyphenolic compounds to effectively scavenge MGO in acidic and alkaline foods.

## 3. Materials and Methods

### 3.1. Materials

Methylglyoxal (MGO, 40% in water), gallic acid (>97.5%), ethyl gallate (≥96.0%), propyl gallate (PG, ≥98%), 2-methylquinoxaline (97%), o-phenylenediamine (98%), diethylenetriaminepentaacetic acid (DETAPAC) (98%), sodium phosphate (Na_2_HPO_4_) (≥99%), sodium hydroxide, hydrochloric acid, formic acid (98%), methanol (gradient grade for LC) were purchased from Sigma-Aldrich (St. Louis, MO, USA). Pyrogallol (>99%) was purchased by TCI (Tokyo Chemical Industry, Tokyo, Japan). Ultrapure water was prepared in a Milli-Q filter system (Millipore, Milan, Italy).

### 3.2. Trapping of MGO by the Phenolic Compounds

Solutions of MGO (1 mM) and the phenolic compounds under investigation (pyrogallol, gallic acid, ethyl gallate, and propyl gallate) (1 mM) were prepared in 200 mM phosphate buffer at the designated pH values (6.5, 7.0, 7.4, and 8.0). Equal volumes of each phenolic compound and MGO solutions were mixed so that the final concentration of the two reactants was 0.5 mM. Samples were incubated at 37 °C in a shaking water bath for 1, 2, 3, 4, and 5 h. The reaction mixture was frozen until MGO analysis. Control experiments were performed with the incubation of MGO in the absence of polyphenolic compounds under the same conditions used in the reactions, in which we did not observe any changes in the MGO concentration, as determined by the o-phenylendiamine derivatization method and HPLC analysis described below.

### 3.3. HPLC-DAD Analysis of MGO

For MGO analysis, the samples were subjected to derivatization with o-phenylenediamine as described previously [1,27]. Briefly, a 500 μL aliquot of supernatant was mixed with 150 μL of 0.2% o-phenylenediamine solution containing 11 mM DETAPAC and 150 μL of 0.5 M sodium phosphate buffer (pH 7). The mixture was immediately filtered through a 0.45 μm syringe filter into an autosampler vial, which was kept at room temperature in the dark for 2 h for derivatization prior to HPLC analysis. The analysis was performed in a Thermo Scientific—Dionex Ultimate 3000 UHPLC system coupled with a Diode Array Detector (DAD). The chromatographic separation was performed on an Agilent C18 column (4.6 × 150 mm, 5 μm) with a C18 guard column (4.6 × 10 mm, 5 μm) using an isocratic mixture of 60% water with 1% formic acid and 40% of methanol with 1% formic acid as the mobile phase at a flow rate of 0.7 mL/min at 25 °C. The injection volume was 10 μL. The signal of 2-methylquinoxaline was used for quantification, and the detection wavelength was at 313 nm. The limit of detection (LOD) and limit of quantitation (LOQ) were 0.06 mg·L^−1^ and 0.17 mg·L^−1^, respectively. Data were collected and integrated with Chromeleon 7.2 software. All measurements were performed in quadruplicate. MGO% trapping was calculated by: $MGO\;trapping\%=\frac{{\left[MGO\right]}_{0}-{\left[MGO\right]}_{t}}{{\left[MGO\right]}_{0}}\times 100$, where [MGO]_0_ corresponds to the concentration of MGO before the onset of the reactions and [MGO]_t_ to the concentration at each time point of the reaction, as determined by the o-phenylendiamine derivatization method and HPLC analysis.

### 3.4. LC-MS Analysis of Reaction Products

To determine the products of the reaction of pyrogallol with MGO, the reaction was carried out as described in Section 2.2, and 500 μL of each sample was placed on ice immediately, and 10 μL of acetic acid was added to them to stop the reaction. Samples were then diluted and analyzed by LC-MS. An Agilent 1260 Infinity series HPLC system (Agilent Technologies, Santa Clara, CA, USA), equipped with an autosampler, a column thermostat, and a binary solvent management system, was utilized for the chromatographic determination. The analysis was performed on an Agilent C18 column (2.1 × 100 mm, 1.8 μm) with a C18 guard column (2.1 × 5 mm, 1.8 μm). The column was eluted with 100% solvent A (100% water with 0.2% acetic acid) for 5 min, followed by linear increases in B (100% methanol with 0.2% acetic acid) to 30% from 5 to 20 min and 80% from 20 to 40 min. The column was then re-equilibrated with 100% A for 5 min. A flow rate and an injection volume of 0.3 mL/min and 10 μL, respectively, were used in all experiments. The column temperature was set at 25 °C. The HPLC system was connected to an Agilent Infinity Mass Spectrometry (MS) 6100 Series system (Agilent Technologies, Santa Clara, CA, USA), equipped with an electrospray interface operating in negative mode polarity. The parameters of the ionization source were as follows: nebulizer gas (nitrogen) pressure, 55 psi; drying gas (nitrogen) flow rate, 12 L/min, drying gas temperature, 350 °C; capillary voltage, 3500 V. Preliminary experiments were performed in full scan mode to identify the main molecular ions and subsequently, selective ion monitoring (SIM) mode was employed. Data acquisition and processing were conducted using OpenLab ChemStation and OriginPro 2023 software.

### 3.5. Statistical Analysis

All measurements were performed in quadruplicate. The results are presented as the mean ± standard deviation of quadruplicates. The error bars in the figures correspond to the standard deviation. Statistical tests were performed using Microsoft Excel and OriginPro 2023. One-way analysis of variance (ANOVA) was applied to the experimental data obtained for each phenolic compound to determine the significance of reaction conditions on MGO-trapping. The significant differences among the means between the reaction conditions for each phenolic compound were estimated through Tukey’s honest significant difference (HSD) test, and *p* < 0.05 was considered statistically significant. The overall data were also tested by means of three-way ANOVA to evaluate the effects of phenolic compound, pH, time, and their interactions on MGO-trapping.

## Data Availability

The original contributions presented in this study are included in the article/Supplementary Materials. Further inquiries can be directed to the corresponding author.

## Supplementary Materials

The following supporting information can be downloaded at: https://www.mdpi.com/article/10.3390/molecules30153086/s1, Table S1: Trapping of MGO (%) by pyrogallol, gallic acid, ethyl gallate, propyl gallate at pH 6.5, 7.0, 7.4 and 8.0 after incubation at the indicated times; Figure S1: Descriptive statistics plots (means plots) obtained by three-way ANOVA showing the effect of each of the three factors examined: polyphenolic compound (a), pH (b) and reaction time (c) on % MGO trapping (Estimate); Figure S2: Descriptive statistics plots (means plots) obtained by three-way ANOVA showing the effect of pair interactions for the three factors examined: polyphenol*pH (a), polyphenol*reaction time (b) and pH*reaction time (c) on % MGO trapping (Estimate); Figure S3: Descriptive statistics plots (means plots) obtained by three-way ANOVA showing the effect for the three factors examined (polyphenol*pH*reaction time) on % MGO trapping; Figure S4: Equilibrium of monohydrated and aldehyde MGO forms.

## Abbreviations

The following abbreviations are used in this manuscript:

MGO	Methylglyoxal
AGEs	Advanced Glycation End Products
RAGE	Receptor for AGEs
LC-MS	Liquid Chromatography-Mass Spectrometry
HPLC	High Performance Liquid Chromatography
DAD	Diode Array Detector
mono-MGO PYR	Mono-MGO conjugated adduct of pyrogallol
di-MGO PYR	Di-MGO conjugated adduct of pyrogallol
DETAPAC	Diethylenetriaminepentaacetic acid
ANOVA	Analysis of Variance
HSD	Honestly Significant Difference
