# Supplementary material for: Effect of pH on the Efficiency of Pyrogallol, Gallic Acid, and Alkyl Gallates in Trapping Methylglyoxal
Source: Molecules. 2025 Jul 23;30(15):3086. doi: 10.3390/molecules30153086 (PMC12348640; doi:10.3390/molecules30153086)
Supplement: Supplementary file 1 [file molecules-30-03086-s001.zip › molecules-3740513-supplementary.pdf]

Supplementary Materials

Effect of pH on the efficiency of pyrogallol, gallic acid and alkyl gallates in trapping methylglyoxal

Haria Hadjipakkou and Eftychia Pinakoulaki*

University of Cyprus, Department of Chemistry

1 Panepistimiou Avenue, 2109 Aglantzia, Nicosia, Cyprus

*Correspondence: effiep@ucy.ac.cy

Table S1. Trapping of MGO (%) by pyrogallol, gallic acid, ethyl gallate, propyl gallate at pH 6.5, 7.0, 7.4 and 8.0 after incubation at the indicated times.

		% MGO trapping			
**Reaction Conditions**		**Pyrogallol**	**Gallic acid**	**Ethyl gallate**	**Ethyl gallate**
pH 6.5	60 min	9.3 ± 1.3^{k}	8.5 ± 1.5^{n}	1.0 ± 0.9^{j}	0.8 ± 0.6^{m}
	120 min	21.2 ± 1.9^{jk}	16.0 ± 2.6^{m}	1.6 ± 1.2^{ij}	2.8 ± 0.7^{lm}
	180 min	32.3 ± 1.9^{ij}	21.4 ± 1.4^{kl}	3.4 ± 0.6^{hij}	3.9 ± 0.9^{klm}
	240 min	43.1 ± 1.8^{hi}	27.5 ± 1.8^{ij}	5.4 ± 0.9^{hij}	5.7 ± 0.8^{jklm}
	300 min	51.7 ± 4.6^{fgh}	31.8 ± 2.0^{hi}	7.3 ± 0.3^{ghi}	8.1 ± 0.8^{ijk}
pH 7.0	60 min	23.3 ± 2.2^{lm}	16.2 ± 1.8^{lm}	1.7 ± 0.2^{ij}	1.1 ± 0.2^{lm}
	120 min	40.6 ± 3.2^{ijk}	27.9 ± 1.9^{ijk}	5.7 ± 0.6^{hij}	3.7 ± 1.4^{klm}
	180 min	$54.2 \pm .53^{ef}$	38.2 ± 1.8^{gh}	10.8 ± 0.5^{fgh}	8.1 ± 1.1^{ijkl}
	240 min	63.9 ± 4.1^{def}	45.4 ± 2.7^{fg}	13.8 ± 2.4^{fg}	12.4 ± 1.6^{hij}
	300 min	70.5 ± 4.4^{cde}	52.5 ± 2.0^{ef}	17.1 ± 0.5^{ef}	16.4 ± 1.7^{gh}
pH 7.4	60 min	30.6 ± 1.6^{klm}	25.2 ± 1.3^{jk}	7.8 ± 2.7^{ghi}	6.1 ± 2.9^{jkl}
	120 min	49.2 ± 1.9^{ghi}	43.4 ± 0.5 g	16.9 ± 1.8^{f}	13.7 ± 3.9^{hi}
	180 min	62.7 ± 2.8^{ef}	55.6 ± 0.2^{e}	26.4 ± 2.6^{e}	20.0 ± 3.4^{fg}
	240 min	70.8 ± 2.8^{cde}	64.1 ± 0.4^{d}	33.5 ± 2.2^{d}	28.2 ± 5.2^{e}
	300 min	76.4 ± 2.3^{bc}	70.1 ± 0.5^{cd}	41.1 ± 2.9^{c}	35.3 ± 3.4^{d}
pH 8.0	60 min	42.0 ± 5.4^{hij}	45.4 ± 3.5^{fg}	23.1 ± 0.8^{e}	28.6 ± 5.4^{ef}
	120 min	62.2 ± 5.6^{ef}	65.1 ± 5.5^{d}	42.1 ± 2.2^{c}	45.9 ± 1.0^{c}
	180 min	74.0 ± 5.8^{bcd}	76.2 ± 5.3^{bc}	55.0 ± 5.4^{b}	60.0 ± 3.5^{b}
	240 min	81.8 ± 5.0^{ab}	82.0 ± 4.9^{ab}	63.3 ± 3.7^{a}	67.5 ± 2.3^{ab}
	300 min	86.9 ± 4.0^{a}	86.6 ± 4.0^{a}	68.7 ± 5.0^{a}	73.2 ± 5.1^{a}

* Data are presented as mean ± standard deviation of quadruplicates. The different letters show statistically significant differences between the reaction conditions (pH, time) for each phenolic compound according to Tukey's HSD test at $p < 0.05$.

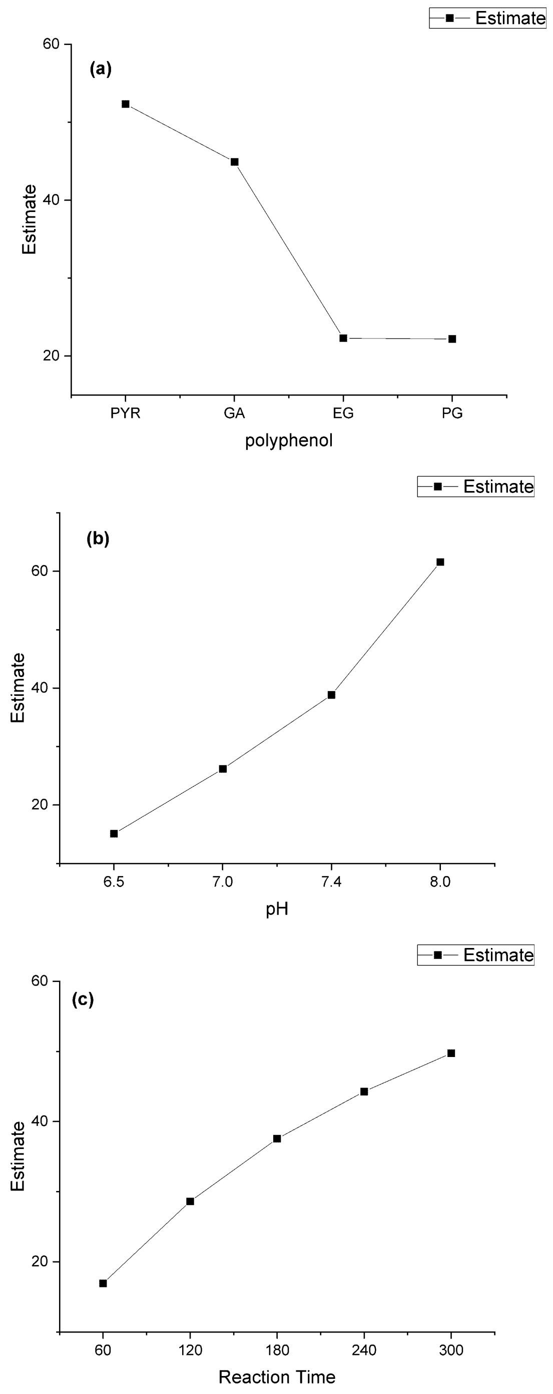

Figure S1. Descriptive statistics plots (means plots) obtained by three-way ANOVA showing the effect of each of the three factors examined: polyphenolic compound **(a)**, pH **(b)** and reaction time **(c)** on % MGO trapping (Estimate).

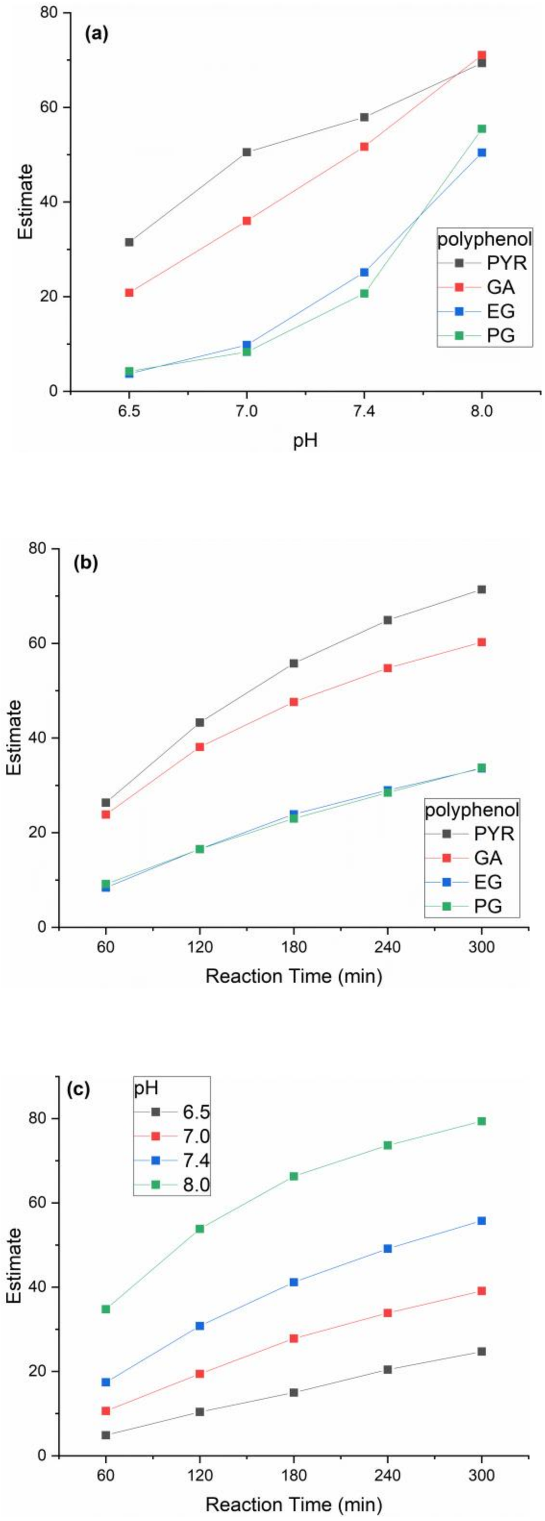

Figure S2. Descriptive statistics plots (means plots) obtained by three-way ANOVA showing the effect of pair interactions for the three factors examined: polyphenol*pH **(a)**, polyphenol*reaction time **(b)** and pH*reaction time **(c)** on % MGO trapping (Estimate).

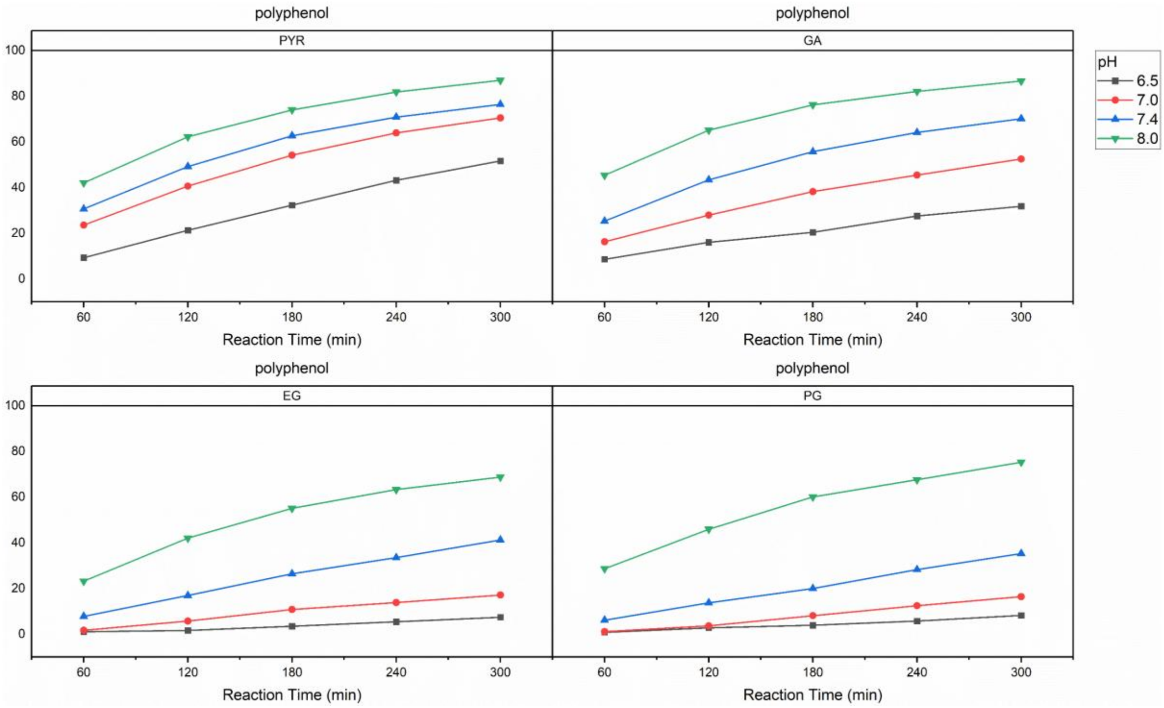

Figure S3. Descriptive statistics plots (means plots) obtained by three-way ANOVA showing the effect for the three factors examined (polyphenol*pH*reaction time) on % MGO trapping.

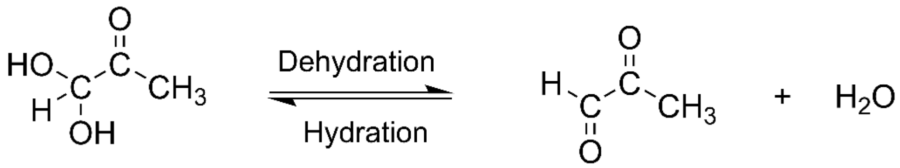

Figure S4. Equilibrium of monohydrated and aldehyde MGO forms.
